# Supplementary material for: Approximating evidence via bounded harmonic means
Source: Stat Comput. 2026 Apr 17;36(3):120. doi: 10.1007/s11222-026-10875-z (PMC13090188; doi:10.1007/s11222-026-10875-z)
Supplement: Supplementary file 1 — (pdf 468 KB) [file 11222_2026_10875_MOESM1_ESM.pdf]

Supplementary Material

Exact Evidence Computation of Rosenbrock Example

Let $\bar{Y} = (\bar{Y}_1, \bar{Y}_2, \ldots, \bar{Y}_d)^\top \in \mathbb{R}^d$ denotes the sample mean of n observations.

Likelihood:

$$\bar{Y}_j \mid \theta \sim \mathcal{N}(\mu_j(\theta), \sigma^2/n), \quad j = 1, 2, \ldots, d$$

where

$$\mu_1(\theta) = \theta_1, \quad \text{and} \quad \mu_j(\theta) = \theta_j + b_{j-1}(\theta_{j-1}^2 - a_{j-1}), \quad j = 2, \ldots, d.$$

The full likelihood density is:

$$p(\bar{Y} \mid \boldsymbol{\theta}) = C \cdot \exp\left(-\frac{n}{2\sigma^2} \sum_{j=1}^{d} (\bar{Y}_j - \mu_j(\boldsymbol{\theta}))^2 \right),$$

where $C = \left(\frac{n}{2\pi\sigma^2}\right)^{d/2}$.

Prior: Flat (improper) prior on all parameters: $\pi(\boldsymbol{\theta}) \propto 1$.

Marginal likelihood: We compute $Z = C \cdot I$, where

$$I = \int_{\mathbb{R}^d} \exp\left(-\frac{n}{2\sigma^2} \sum_{j=1}^{d} (\bar{Y}_j - \mu_j(\boldsymbol{\theta}))^2 \right) d\boldsymbol{\theta}.$$

Change of variables: Define $\boldsymbol{\phi} = g(\boldsymbol{\theta})$ by $\phi_j = \mu_j(\boldsymbol{\theta})$ for all j:

$$\phi_1 = \theta_1, \quad \phi_j = \theta_j + b_{j-1}(\theta_{j-1}^2 - a_{j-1}) \quad \text{for } j = 2, \ldots, d.$$

The Jacobian matrix $J = \frac{\partial \boldsymbol{\phi}}{\partial \boldsymbol{\theta}}$ is lower bidiagonal:

$$J = \begin{pmatrix} 1 & 0 & 0 & \cdots & 0 \\ 2b_1\theta_1 & 1 & 0 & \cdots & 0 \\ 0 & 2b_2\theta_2 & 1 & \cdots & 0 \\ \vdots & \ddots & \ddots & \ddots & \vdots \\ 0 & \cdots & 0 & 2b_{d-1}\theta_{d-1} & 1 \end{pmatrix}.$$

Since J is lower triangular with all diagonal entries equal to 1, we have $\det(J) = 1$ for all $\boldsymbol{\theta} \in \mathbb{R}^d$.

Evaluation: With $|\det(J)| = 1$, the change of variables gives $d\boldsymbol{\theta} = d\boldsymbol{\phi}$, and the integral becomes:

$$I = \int_{\mathbb{R}^d} \exp\left(-\frac{n}{2\sigma^2}\sum_{j=1}^{d}(\bar{Y}_j - \phi_j)^2\right) d\boldsymbol{\phi} = \prod_{j=1}^{d}\int_{-\infty}^{\infty} \exp\left(-\frac{n}{2\sigma^2}(\bar{Y}_j - \phi_j)^2\right) d\phi_j.$$

Each integral equals $\sqrt{2\pi\sigma^2/n}$, so $I = \left(\frac{2\pi\sigma^2}{n}\right)^{d/2}$. Therefore:

$$Z = C \cdot I = \left(\frac{n}{2\pi\sigma^2}\right)^{d/2} \times \left(\frac{2\pi\sigma^2}{n}\right)^{d/2} = 1.$$

Additional Results of Rosenbrock Example

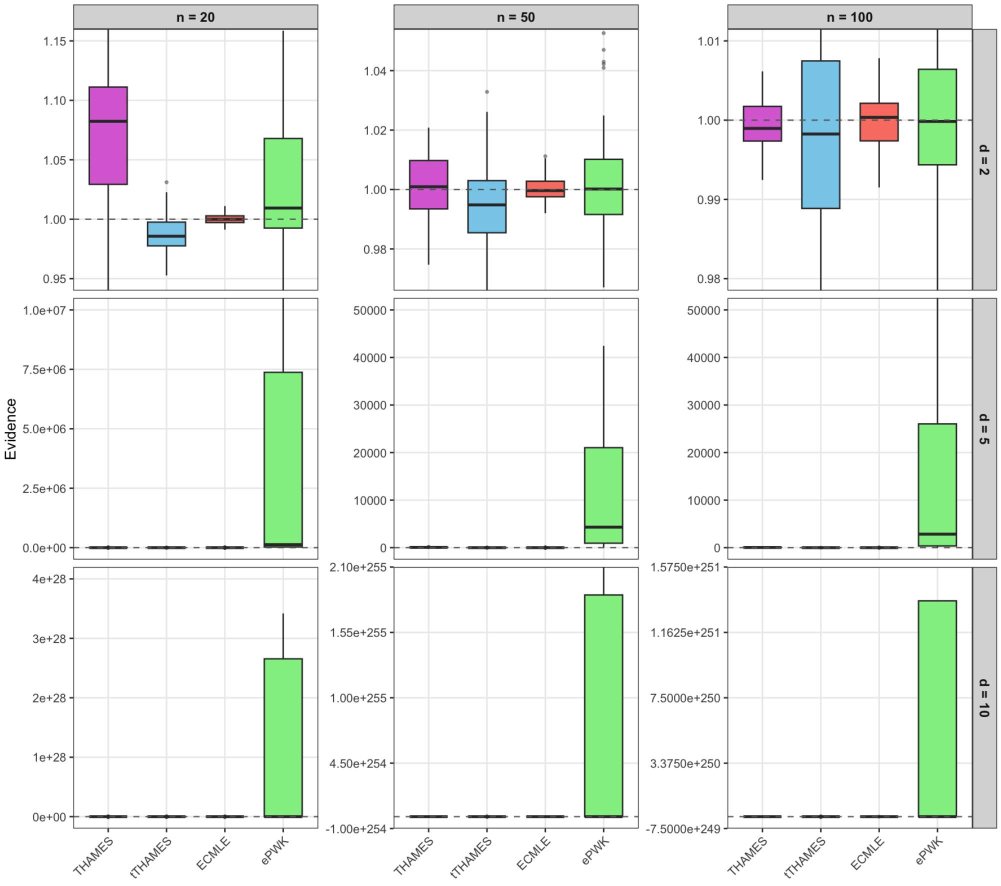

Figure 12: (**Example 3**) Boxplots of marginal likelihood estimates for the Rosenbrock distribution posterior in dimensions $d \in \{2, 5, 10\}$ and sample sizes $n \in \{20, 50, 100\}$, based on $M = 100$ independently generated datasets. The y-axis scale is adjusted to show that ePWK produces estimates far outside the acceptable range, particularly in higher dimensions. For each dataset, all methods use the same posterior sample, but with method-specific numbers of draws chosen so that all methods operate under a comparable computational budget.

.

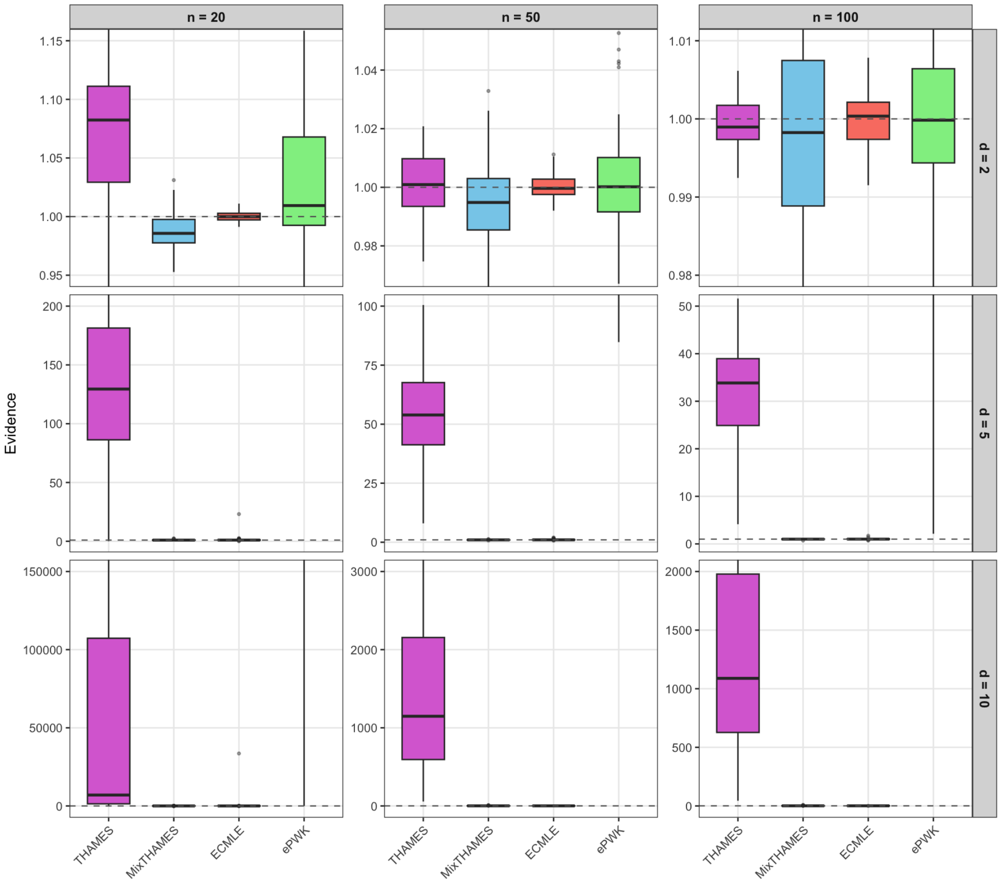

Figure 13: (**Example 3**) Boxplots of marginal likelihood estimates for the Rosenbrock distribution posterior in dimensions $d \in \{2, 5, 10\}$ and sample sizes $n \in \{20, 50, 100\}$, based on $M = 100$ independently generated datasets. The y-axis scale is adjusted to show that THAMES produces estimates far outside the acceptable range, particularly in higher dimensions. For each dataset, all methods use the same posterior sample, but with method-specific numbers of draws chosen so that all methods operate under a comparable computational budget.
